# Necrotizing Fasciitis Presenting as Generalized Weakness, Malaise, and Acute Kidney Injury

**DOI:** 10.7759/cureus.31674

**Published:** 2022-11-19

**Authors:** Laurence Stolzenberg, Alexis Koch, Austin Huang, Mohammad Usman, Jason R Seale

**Affiliations:** 1 Orthopedic Surgery, Alabama College of Osteopathic Medicine, Dothan, USA; 2 Pediatric Medicine, Alabama College of Osteopathic Medicine, Dothan, USA; 3 Neurology, Alabama College of Osteopathic Medicine, Dothan, USA; 4 Anesthesiology, Alabama College of Osteopathic Medicine, Dothan, USA; 5 General Surgery, Decatur Morgan Hospital, Decatur, USA

**Keywords:** bacteroides fragilis, wound vacuum, surgical debridement, general weakness, malaise, acute kidney injury, cellulitis, fournier gangrene, necrotizing fasciitis

## Abstract

Necrotizing fasciitis (NF) is a surgical emergency that must be diagnosed promptly in order to avoid serious consequences or death. Additionally, symptoms of this condition are similar to less severe skin and soft tissue infections such as cellulitis or erysipelas and can be easily confused. In this case, the patient presented to the emergency department with systemic symptoms, notably malaise and generalized weakness. A cutaneous complaint, a “labial cyst”, was only elicited after more specific questioning. Laboratory investigations revealed abnormal renal function tests (RFTs), suggestive of an acute kidney injury. An abdominal/pelvic computed tomography (CT) showed gas in the subcutaneous tissue. These findings led to clinical suspicion of NF, prompting a general surgery consultation. The surgeon proceeded to perform extensive debridement following the discovery of necrotic tissue. The prompt diagnosis and treatment of this condition resulted in patient survival and expected recovery. It is, therefore, critical to keep this condition in mind when diagnosing apparent skin and soft tissue infections presenting with abnormal RFTs due to the possibility of rapid decline and death if the NF is left untreated. Additionally, this is a case of less frequent Fournier's gangrene in a non-diabetic female. Finally, it underlines the importance of eliciting additional symptoms, even those that may seem unrelated, or less concerning, to the patient’s initial complaint.

## Introduction

Necrotizing fasciitis (NF) is a skin and soft tissue infection characterized by necrosis. It is known to cause rapid local tissue destruction in addition to tissue death [1]. The initial presentation of NF, according to existing literature, is typically fever, pain, tenderness, swelling, and erythema [1-3]. This leads to difficulty in the initial recognition and diagnosis of NF as its clinical presentation is similar to that of cellulitis and erysipelas [1].

NF has multiple predisposing conditions, the most common of these are diabetes mellitus (DM) (44.5%), trauma (31.4%), obesity (28.3%), liver disease and alcoholism (15.1%), peripheral vascular disease (13.6%), and surgical wounds (4.3%) [2]. NF of the perineal region, referred to as Fournier’s gangrene, is associated with 12.6% of cases, the majority of these in men [2]. 

Presumptive diagnosis is often made based on clinical presentation with signs that may include swelling (80.8%), localized pain (79%), and positive wound culture (76.5%). Additionally, habitual signs of severe infection such as fever (40%), hypotension (24.8%), and gas on x-ray (24.8%), may be present but are less common [2]. The final diagnosis is made during surgery based on the presence of extensive necrotic tissue extending to fascia. Treatment is usually emergent surgical resection, broad-spectrum antibiotics, and, when available, hyperbaric oxygen treatment [4,5]. 

Necrotizing soft tissue infections are quite rare, but they progress rapidly and have a high incidence of mortality, which requires a high index of suspicion, rapid diagnosis, and treatment. One of the most significant complications is life-threatening sepsis and septic shock [6]. The other complications are disseminated intravascular coagulation (DIC), amputation of one or multiple limbs, organ dysfunction, and death. The overall mortality rate varies significantly based on comorbid conditions and speed of diagnosis but is approximately 11-22% in the United States (US) [7].

A reduction of renal function within a short period of time, days or weeks, is termed an acute kidney injury (AKI) [8]. Recognized biomarkers for this disorder include an increase in serum concentration of urea nitrogen and creatinine [8,9]. This disorder has many causes, which most literature categorizes into pre-renal causes (such as hypovolemia), intrarenal causes, and postrenal causes [8,10]. There are several diagnostic criteria including either an increase in serum creatinine (sCr) of at least 0.3mg/dL within 48 hours or a 50% increase in sCr from baseline in seven days [8,9]. this condition is quite common, being associated with eight out of every 10,000 admissions in the US [8]. 

## Case presentation

A 77-year-old Caucasian female presented to the Emergency Department with a chief complaint of constant lower abdominal pain, generalized weakness, and malaise for two days. After further questioning, she also mentioned a “cyst on her labia that was bothering her.” The patient denied any improvement or worsening of symptoms. She had several underlying medical conditions including hypertension, hypothyroidism, hyperlipidemia, and asthma. She had confirmed allergies to clindamycin, penicillin, and butalbital, all of which caused nausea and vomiting. No pertinent family history was elicited during the interview. A review of systems was positive for malaise, abdominal pain, genitalia pain, and generalized muscle weakness. Her body mass index (BMI) was 32.3kg/m^2^. Vital signs revealed a temperature of 99°F, a pulse rate of 72 beats per minute, a blood pressure of 136/41 mmHg, and an oxygen saturation of 93%. Physical examination revealed an alert woman with clear lungs and normal cardiovascular examination including normal peripheral pulses and abdomen with mild diffuse tenderness throughout all four quadrants. This examination also revealed a red, indurated, warm, and tender right perineum and labia majora with no observed drainage, ulcerations, necrosis, or open wounds. There was no apparent crepitus. 

**Figure 1:**
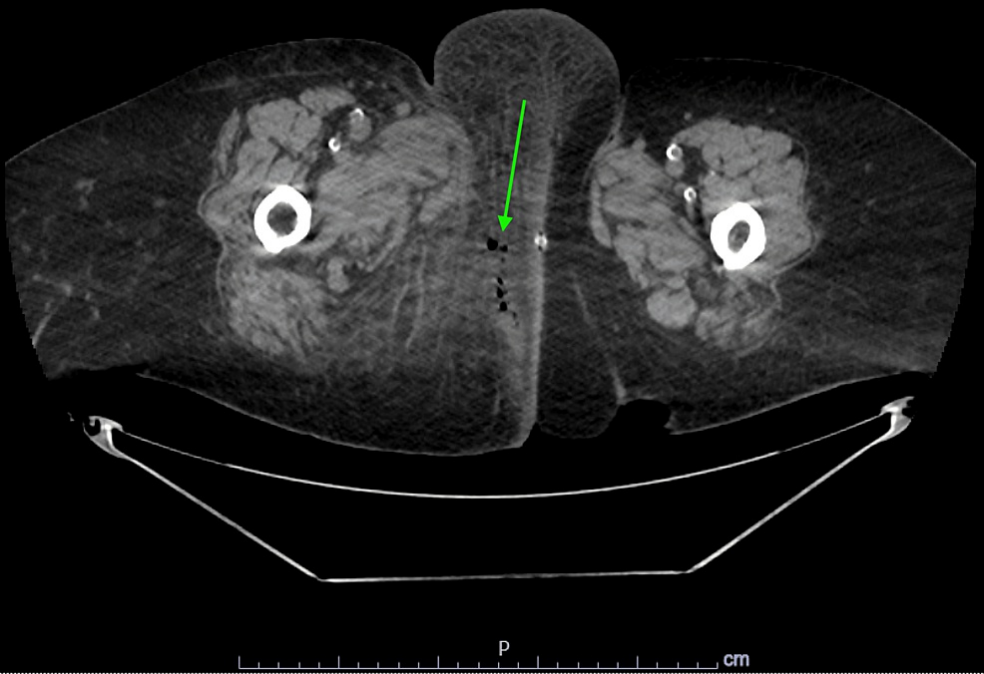
CT scan (axial cut) showing right-sided gas in the connective tissue.

**Figure 2:**
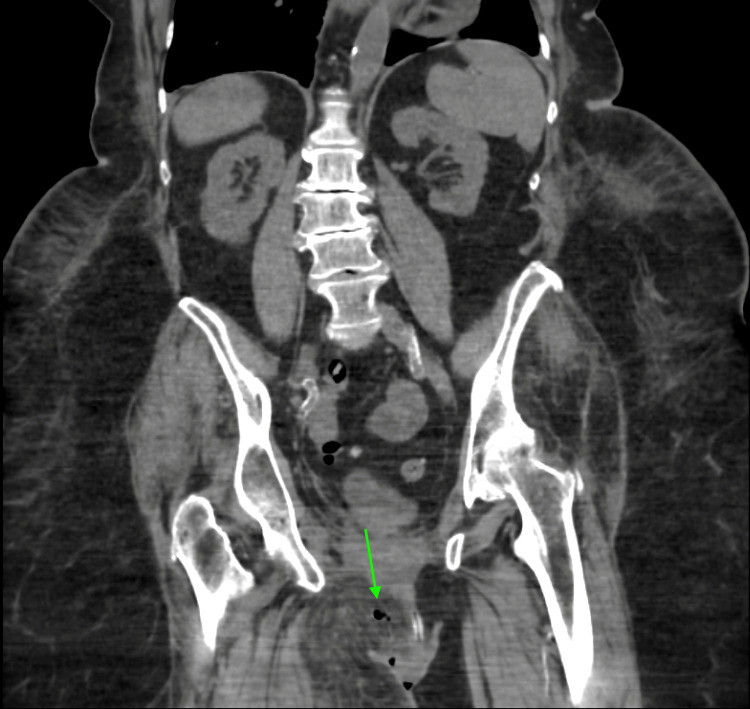
CT scan (coronal cut) showing right-sided gas in the connective tissue.

At this point, the differential included a low but present suspicion for acute coronary syndromes (ACS), along with the more evident skin or soft tissue infections including possible necrotizing fasciitis. Imaging and laboratory workups were ordered. Initial laboratory values were notable for blood urea nitrogen (BUN) of 56 mg/dL and creatinine of 3.2 mg/dL, significantly outside the patient’s baseline. Other notable values were high sensitivity troponin of 62 ng/L, although this had been elevated during previous visits, and pro-brain natriuretic peptide of 3290 pg/mL. Her white cell count remained at the high end of normal at 10,700 white blood cells per microliter. Her hemoglobin was at 9.2 g/dL, whereas her baseline had been around 12.9 g/dL during a previous visit. A urinalysis was unrevealing, and her sodium was within normal limits. Glucose was 108 mg/dL. At this point, a CT scan of the abdomen and pelvis was ordered due to the abdominal pain of unclear etiology and uncertain skin signs. The CT showed perineal cellulitis and a small volume of free gas in the right perineal subcutaneous tissues (Figures 1, 2). 

This raised clinical suspicion of NF and the department of general surgery was consulted. Based on the physical examination and imaging, the surgeon consulted had a strong suspicion for NF. Consequently, the surgeon decided to perform an emergent incision and drainage in the operating room under general anesthesia. The patient had already been put on broad-spectrum antibiotics prior to surgery. These antibiotics were cefepime 1g intravenously (IV) and vancomycin 1g IV. The patient was taken to the operating room. An initial incision was made over the right ischiorectal and buttock skin and carried down into the ischiorectal space where necrotic tissue was encountered. The surgeon and the medical student continued to debride a significant amount of necrotic tissue including skin, subcutaneous fat, and fascia. The surgeon described the wound as having a foul odor and “dishwater drainage.” The area debrided was 22cm long by 12cm wide by 4cm deep. This is when the final diagnosis of NF was made. 

The debrided wound was left open for secondary healing owing to contamination and packed with hypochlorous acid-saturated gauze. The patient recovered from anesthesia without apparent complications, other than pain and an intraoperative blood loss of approximately 100mL. Following surgery, the culture showed a predominant Gram-negative anaerobe, *Bacteroides fragilis*. Accordingly, considering the patient's allergies and the information available for the bacterium, cefepime was replaced with meropenem 1g IV four times a day. She continued to recover over the next few days. A wound vacuum was subsequently installed to speed up healing by second intention. On the 10th day post admission, the patient was discharged and transferred to a specialized long-term acute care (LTAC) facility, where she continues along the path to full recovery. She was told to follow up weekly with the surgeon in the hospital’s outpatient wound care clinic, after her discharge from LTAC, until full wound closure. 

## Discussion

This patient had a relatively atypical presentation for NF. This affirmation is based on the tool created to assist clinicians in establishing levels of suspicion and risk for NF called the Laboratory Risk Indicator for Necrotizing Infection (LRINEC) [11,12]. Laboratory findings for this patient were significant for anemia and elevated sCr and BUN. They were also notable due to the absence of hyponatremia, hyperglycemia, and leukocytosis. The previous values are important components of assessing a patient with suspected NF as they are each part of the LRINEC [11,12]. Based on available laboratory data as mentioned above, our patient scored a four on this protocol, whereas a six would be considered significant [11,12]. The accepted guidelines did suggest continuing with serial labs and broad-spectrum antibiotics [2,11,12]; watchful waiting may have led to a worse outcome for this patient. The point that stood out most when using the LRINEC for this patient was her unremarkable white cell count. While we may expect an elevated white blood cell count in a patient with such a severe infection, it was absent in this case. This could be explained by the patient’s age and limited overall capacity to mount a robust immune reaction [13] or simply that the infection was caught early in its course. Looking back, it would have been interesting and indicative to see inflammatory markers for this patient [12]. Finally, as detailed in the introduction, the perineal region is affected in only 12.6% of cases, most of which occur in males [2], and the lack of either DM or trauma all come together to make this a less common presentation of NF.

One of the possible diagnostic criteria for AKI is a 50% or greater increase in sCr from baseline in seven days [8-10]. The last renal function tests (RFTs) for this patient had been taken three months ago, revealing a BUN of 36mg/dL and sCr of 1.7mg/dL. At admission, her RFTs showed BUN of 56mg/dL and sCr of 3.2 mg/dL, revealing an increase of over 85%. This allowed a tentative and clinical diagnosis of AKI in this patient, even though it was impossible to know exactly when the rise in creatinine occurred and the rate at which it did. The exact cause cannot be confirmed, as any number of events or pathological processes could have occurred in the interim unknown to the clinicians. Nonetheless, multiple clinicians agreed based on available documentation associating necrotizing soft-tissue infections (NSTIs) with AKI [14], the significant variation between her baseline and current RFTs, and a review of her available medical history showed a lack of other significant changes in underlying medical conditions. A review of available literature demonstrates that NSTIs are associated with AKI, due to multiple possible underlying pathological processes caused by the infection, including hypoperfusion, rhabdomyolysis, and acute tubular necrosis amongst many other listed causes [14]. The learning point here is simply a further case to reinforce the link between NSTIs and AKI, as well as the importance of following RFTs during the clinical course of a case of NF to prevent serious renal repercussions.

According to available literature, patients with NF will often present with excruciating pain out of proportion to presenting symptoms and sepsis [2,5,11]. Contrary to this expected presentation, our patient admitted only discomfort and no frank pain at the location of the lesion. This may have been due to the rapid progression and subsequent damage of local nerve endings, which is not unheard of during serious infection [15]. Another possibility for the lower reported pain from this patient may be a high pain tolerance. As this patient presented to the ED with her biological children and grandchildren, we can consider the well-documented fact that multiparous women have a higher pain threshold [16]. Whatever the reason, we can see that it is important to probe further and consider this diagnosis even in the absence of pain out of proportion to the lesion.

Additionally, there are frequent mentions in the literature of physical examinations for patients with NF demonstrating crepitus [1,3,11]. Another important point in the discussion of our patient is that there was no palpable crepitus, although further review showed this expected sign only actually occurs in 4.9% of cases [17]. The point here is that crepitus is rare in NF and should not be considered a requirement for diagnosis.

## Conclusions

We presented the case of a patient appropriately diagnosed and treated for NF that had inconclusive presenting symptoms of malaise, weakness, abdominal pain, and a skin lesion that was initially considered less pressing to the patient. This case of NF also included significant renal involvement, which is mentioned in the literature, but very infrequently. The available laboratory workup was also somewhat atypical compared to existing literature, notable for lack of significant leukocytosis or hyperglycemia. We also hope this case will reinforce the need to probe further issues and other symptoms, even those the patient themselves may consider unimportant to their initial complaint. Finally, we also wish to bring greater awareness of the possibility, although less common, of Fournier's gangrene in non-diabetic female patients. Finally, considering the high morbidity and mortality that can result from NF, early diagnosis and aggressive treatment are essential.
